# [Retracted] MicroRNA‑195 inhibits the behavior of cervical cancer tumors by directly targeting HDGF

**DOI:** 10.3892/ol.2024.14597

**Published:** 2024-07-29

**Authors:** Rongrong Song, Lin Cong, Guantai Ni, Min Chen, Honmei Sun, Yunxia Sun, Meiling Chen

Oncol Lett 14: 767–775, 2017; DOI: 10.3892/ol.2017.6210

Following the publication of this paper, it was drawn to the Editor's attention by a concerned reader that certain of the Transwell cell migration and invasion assay data shown in Fig. 5C on p. 773 were strikingly similar to data that had appeared in different form in different articles written by different authors that were under consideration for publication at around the same time, several of which have subsequently been retracted.

Owing to the fact that the contentious data in the above article were already under consideration for publication prior to its submission to *Oncology Letters*, the Editor has decided that this paper should be retracted from the Journal. The authors were asked for an explanation to account for these concerns, but the Editorial Office did not receive a reply. The Editor apologizes to the readership for any inconvenience caused.

